# Photosynthetically Controlled Spirulina, but Not Solar Spirulina, Inhibits TNF-α Secretion: Potential Implications for COVID-19-Related Cytokine Storm Therapy

**DOI:** 10.1007/s10126-021-10020-z

**Published:** 2021-02-10

**Authors:** Asaf Tzachor, Or Rozen, Soliman Khatib, Sophie Jensen, Dorit Avni

**Affiliations:** 1 Centre for the Study of Existential Risk & Cambridge Global Food Security Research Center, University of Cambridge, Cambridge, UK; 2 Sphingolipids, Active Metabolites and Immune Modulation Laboratory, MIGAL – Galilee Research Institute, Kiryat Shemona, Israel; 3 Natural compounds and analytical chemistry Laboratory, MIGAL - Galilee Research Institute and Tel Hai college, Kiryat Shemona, Israel; 4 MATIS – Food and Biotech Research and Development, Reykjavík, Iceland

**Keywords:** COVID-19, Cytokine storm, Immunology, Biotechnology, Spirulina, Anti TNF-α therapy

## Abstract

An array of infections, including the novel coronavirus (SARS-CoV-2), trigger macrophage activation syndrome (MAS) and subsequently *hypercytokinemia*, commonly referred to as a cytokine storm (CS). It is postulated that CS is mainly responsible for critical COVID-19 cases, including acute respiratory distress syndrome (ARDS). Recognizing the therapeutic potential of Spirulina blue-green algae (*Arthrospira platensis*), in this in vitro stimulation study, LPS-activated macrophages and monocytes were treated with aqueous extracts of Spirulina, cultivated in either natural or controlled light conditions. We report that an extract of photosynthetically controlled Spirulina (LED Spirulina), at a concentration of 0.1 µg/mL, decreases macrophage and monocyte-induced TNF-α secretion levels by over 70% and 40%, respectively. We propose prompt in vivo studies in animal models and human subjects to determine the putative effectiveness of a natural, algae-based treatment for viral CS and ARDS, and explore the potential of a novel anti-TNF-α therapy.

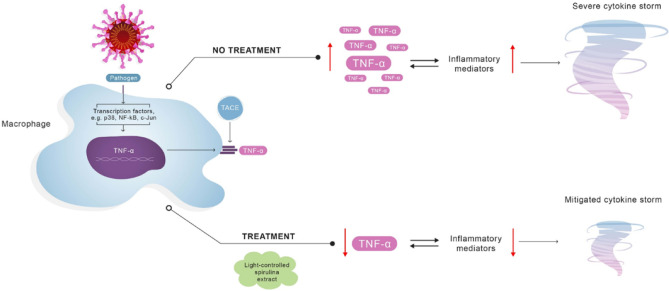

## Introduction

The novel coronavirus disease (COVID-19) is an emerging contagious respiratory tract illness caused by the severe acute respiratory syndrome coronavirus 2 (SARS-CoV-2) (Hu et al. 2020). From late 2019, SARS-CoV-2 infections have resulted in an array of clinical responses which differ between individual cases, from asymptomatic conditions to detrimental manifestations and mortality. Whereas precise immune-pathological processes that COVID-19 activates remain, as of yet, contested (Coperchini et al. 2020), there is an ostensible agreement on the major mechanism by which the virus causes severe symptoms.

Epidemiological studies have indicated that exposure to the etiologic agent SARS-CoV-2 provokes macrophages and monocytes to release an excessive amount of different pro-inflammatory cytokines, such as tumor necrosis factor (TNF)-α and interleukin (IL)-6, to cause a *hypercytokinemia*, commonly referred to as a cytokine storm (CS) (Ishikawa 2012; Ye et al. 2020).

An influx of TNF-α, as part of the CS, destabilizes endothelial cell networks and induces damage of vascular barrier, capillary damage, diffuse alveolar damage (DAD), apoptotic cell death, and multi-organ failure. Furthermore, a recent analysis indicated higher systemic levels of IL-2, IL-7, IL-10, monocyte chemoattractant protein-1 (MCP-1), macrophage inflammatory protein-1A (MIP-1A), and TNF-α, among critically ill COVID-19 patients (McGonagle et al. 2020; Ruan et al. 2020).

Specifically, excess release of TNF-α plays a critical role in disrupting the lung endothelial and epithelial barriers, which may cause acute respiratory distress syndrome (ARDS) (Shimizu 2019). ARDS requires admission to intensive care units (ICU), where invasive mechanical ventilation may be administered (Mittermaier et al. 2020). Furthermore, it is generally accepted that ARDS is the main cause of death of patients with COVID (Mehta et al. 2020). The prevalence of COVID-19 ARDS incidences led to a global public health emergency, prompting governments to suspend social activities and impose unprecedented movement restriction and social distancing. These measures were set to maintain ICUs within their operational capacity limitations, a pending public healthcare concern (Alkuzweny et al. 2020; Moghadas et al. 2020).

Considering the role of TNF-α in triggering COVID-19-related cytokine storm syndrome (COVID-CS) and ARDS, it is necessary to develop new approaches for anti-TNF therapy. Indeed, since the outbreak of the pandemic, TNF-α blockers have shown promising outcomes in treating, and mitigating, severe illness (Robinson et al. 2020).

Herein, a novel approach is proposed for TNF-α inhibition, based on a treatment with the blue-green algae Spirulina (*Arthrospira platensis*) extract.

The potential health benefits of Spirulina are well documented (Belay et al. 1993; Furmaniak et al. 2017). This blue-green algae contains C-phycocyanin (C-PC), a pigment-binding protein, which enhances antioxidation, anti-inflammation, and anti-tumor activities (Cian et al. 2012; Saini and Sanyal 2015). Furthermore, Spirulina may be cultivated in different conditions and extracted using various techniques, which may affect the bioactive metabolite content of Spirulina (Minhas et al. 2016). Under certain conditions, for instance, irradiation by light-emitting diodes (LED) to control photosynthesis, algal bioactivity such as anti-inflammatory properties may be enhanced (De Morais et al. 2015; Ooms et al. 2016).

In this study, we exposed macrophages and monocytes activated by the pathogenic stimulator lipopolysaccharide (LPS) to different doses of Spirulina extracts, cultivated in either full-range solar spectrum or controlled light conditions. We report that an aqueous extract of a photosynthetically controlled Spirulina (LED Spirulina) inhibits TNF-α secretion by over 70% from LPS-activated macrophages and over 40% from LPS-activated monocyte cells.

## Materials and Methods

### Reagents

Cell lines for measuring anti-inflammatory activity human monocyte (THP-1) and murine macrophages (RAW 264.7) were used. All were provided by the American Type Culture Collection (ATCC, USA). Additionally, Rosewell Park Memorial Institute (RPMI) 1640, Dulbecco’s Modified Eagle Medium (DMEM) were purchased from ATCC. Fetal bovine serum (FBS), penicillin-streptomycin, sodium pyruvate, and glutamine were purchased from Biological Industries (Beit HaEmek, Israel). 2-Mercaptoethanol was purchased from BIO-RAD and ELISA kits were purchased from PeproTech. LPS was purchased from Sigma-Aldrich (Sigma-Aldrich, Israel).

### Culture and Extraction

Spirulina (UTEX 3086) was cultivated in Zarrouk medium (Rajasekaran et al. 2016). Cultivation was carried out in 180-L flat panel airlift photobioreactors. The cultures were maintained under agitation induced by insufflation of filtered air, with flow aeration of 0.5 vvm (air volume/medium volume/minute).

Temperature was kept at 31 ± 2 °C and pH was maintained at 10.8 ± 0.2 °C. Cultures were grown under two illumination conditions: (A) full-range solar spectrum at irradiance of 750 μmol/(m^2^s), here referred to as “Solar Spirulina”, and (B) red/blue/UV at irradiance of 750 μmol/(m^2^s) (USP # 63/026,764), here referred to as “LED Spirulina”.

The irradiance was measured on the surface of the flask using a LI-250A light meter (Nebraska, USA) and a Li-Cor quantum sensor. Both cultures were water-extracted using physical freeze-thawing for cellular disruption (Chu et al. 2010), obtaining one Solar Spirulina extract and one LED Spirulina extract.

### Metabolomics Profile of the Full Spectrum and LED Spirulina Extracts

Solar and LED Spirulina extracts were dissolved in methanol (100 $\mu g$ /mL) and 5 $\mu$L of the solutions were injected into a UHPLC connected to a photodiode array detector (Dionex Ultimate 3000), with a reverse-phase column (ZORBAX Eclipse plus C18, 3.0×100°mm, 1.8°μm).

The mobile phase consisted of (A) DDW with 0.1% formic acid and (B) acetonitrile containing 0.1% formic acid. The gradient started with 2% B then increased to 30% B in 4 min, then increased to 40% B in 1 min and kept isocratic at 40% B for 3 min. Then increased to 98% B in 6 min and kept isocratic at 98% B for 9 min.

Phase B returned to 5% in 3 min and the column allowed to equilibrate at 5% B for 5 min before the next injection. The flow rate was 0.4 mL/min. MS/MS analysis performed with heated electrospray ionization (HESI-II) source connected to a Q Exactive™ Plus Hybrid Quadrupole-Orbitrap™ Mass Spectrometer Thermo Scientific™. ESI capillary voltage was set to 3500 V, capillary temperature to 300 °C, gas temperature to 350 °C, and gas flow to 35 mL/min.

The mass spectra (m/z 67–1000) acquired in negative and positive-ion mode with high resolution (FWHM = 70,000). Data-dependent MS/MS was performed with collision energy of 15, 50, and 100 eV**.**

### Measurement of the Secretions of Pro-inflammatory Cytokines from RAW264.7 Macrophages

The amounts of TNF-α and IL-6 secreted from mouse macrophage cells were measured with TNF-α and IL-6 ELISA kits (PeproTech) according to manufacturer instructions. First, RAW264.7 cells (1 × 10^5^ cells/well) were inoculated into a 96-well plate and cultured for 48 h in DMEM 5% FBS (*v*/*v*) and penicillin-streptomycin (100 IU/mL and 1 µg/mL) at 37 °C with 5% CO_2_. Various concentrations of the Spirulina extracts were added 20 min prior to LPS (100 ng/mL) stimulation for additional 4 h of culturing. The concentrations of the cytokines were measured in the ELISA reader infinite M200 PRO (TECAN, Switzerland) at 450 nm with correction at 620 nm.

### Measurement of the Secretions of Pro-inflammatory Cytokines from Human THP-1 Monocyte Cells

The amount of TNF-α secreted from human monocyte cells was determined with TNF-α ELISA kit (PeproTech) according to manufacturer instructions. THP-1 cells (1 × 10^5^ cells/well) were inoculated into a 96-well plate and cultured for 48 h in RPMI 1640 culture medium supplemented with 10% (*v*/*v*) heat-inactivated FBS, 1% sodium pyruvate (11.0 mg/mL (100 mM)), 0.05 µM 2-mercaptoethanol, and 1% penicillin-streptomycin (100 IU/mL and 1 µg/mL) at 37 °C with 5% CO_2_. Various concentrations of Spirulina extracts were added 20 min prior to LPS (100 ng/mL) stimulation for additional 2 h of culturing. The concentrations of the cytokines were measured in the ELISA reader infinite M200 PRO (TECAN, Switzerland) at 450 nm with correction at 620 nm.

### Data Preprocessing

Peak annotation and peak area integration performed with Compound Discoverer 3.1 (Thermo Xcalibur, Version 3.1.0.305). Compound putative identification was performed based on MS spectral data using MzCloud database and HRMS and isotope profile data using ChemSpider database.

### Statistical Analysis

All in vitro experimental data was obtained from three separate experiments performed in three replicates, and statistical significance was determined by one-way analysis of variance (ANOVA) and post hoc Tukey, using the Statistical Analysis System program, PRISMA 8. The difference between the significance levels was set to *p* < 0.05.

## Results

### Metabolomics Profile from Spirulina Extracts of Two Different Cultivation Conditions

**Fig. 1:**
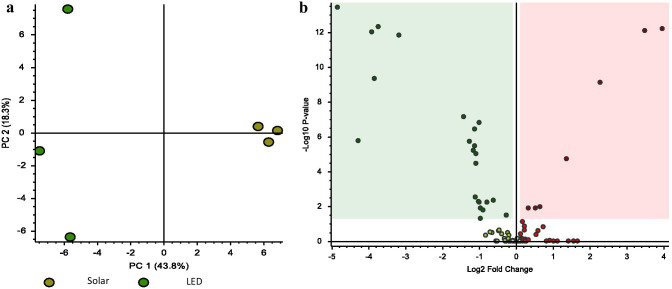
Multivariate statistical analyses. The principal component analysis (PCA) score plots of the Solar and LED Spirulina extracts (**a**). Differential analysis that presents all the difference of the metabolite profile between Solar and LED Spirulina extracts (**b**). Dots in red are the upregulated compounds in the LED extract compared with Solar extract. Dots in green are the downregulated compounds in the LED extract compared with Solar extract. Squared metabolites are significantly increased (red) or decreased (green) at the LED extract compared with Solar extract with *P* value < 0.05 using *t* test statistical analysis

The metabolomics profile from Spirulina extracts was determined using un-targeted LC/MS/MS. PC analysis of the 109 detected features (metabolites) is displayed in Fig. 1a. The first two principal components of the PCA score plot were responsible for 62.1% (43.8% for PC1 and 18.3% for PC2) of the overall variance of the metabolomics profile, showing a clear separation between the Solar and LED Spirulina extracts. Seven compounds were significantly upregulated in the LED extract compared with Solar extract while 23 compounds downregulated (Fig. 1b). Two of the upregulated compounds, sorbitol and adenosine derivate, annotated based on MS/MS spectra using mzCloud database with MS/MS spectral similarity of 97% and 91%, respectively. These bioactive compounds significantly increased in the LED extract by 1.7- and 4.8-fold with *P* value of 0.01 and 7.8×10^–10^, respectively.

In addition, C-phycocyanin (CPC) bioactive compound was also significantly increased in the LED extract, by 4.7-fold.

### Inhibition of Pro-inflammatory Cytokine Secretion by the Extracts

Monocytes and macrophages play a pivotal role in COVID-related CS; therefore, the inhibitory effect of the extracts related to CS was tested in both macrophages and monocyte cells.

**Fig. 2:**
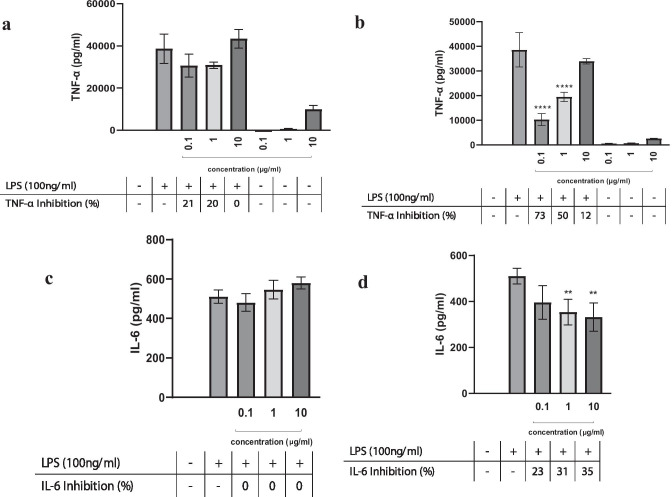
The secretion of TNF-α or IL-6 from LPS-induced RAW264.7 cells after treatment with various concentrations of Solar (**a**, **c**) and LED (**b**, **d**) Spirulina extracts. RAW264.7 were incubated with the indicated doses of spirulina extract in the presence or absence of LPS for 4 h. TNF-α and IL-6 secretion were determined by ELISA. Values are presented as means ± SD; **p < 0.01, ****p < 0.001 and compared with the LPS group

Our results illustrate the effects of the two extracts (Solar Spirulina and LED Spirulina) on LPS-induced secretion of TNF-α from mouse macrophages. LED Spirulina shows a 70% reduction in TNF-α levels at the concentration of 0.1 μg/mL (Fig. 2b), while no inhibitory effect was observed for Solar spirulina (Fig. 2a). In contrast to the inhibitory effect on TNF-α, LED Spirulina had no effect on the secretion of IL-6 at 0.1 μg/mL (Fig. 2d). Moreover, results indicate that at higher dose of LED Spirulina extract (10 μg/mL), there was a minor effect on IL-6 secretion (Fig. 2d), while no inhibitory effect was observed on TNF-α (Fig. 2b). Solar Spirulina had no effect on IL-6 secretion (Fig. 2c).

**Fig. 3:**
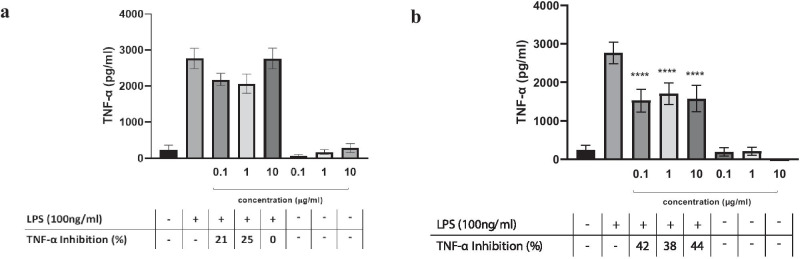
The secretion of TNF-α from LPS-induced THP-1 human monocyte cells by the treatment of various concentrations of the Solar (**a**) or LED Spirulina (**b**) extracts. THP-1 cells were incubated with the indicated dose of Spirulina in the presence or absence of LPS for 2 h. TNF-α secretion was determined by ELISA. Values are presented as means ± SD; ****p < 0.001 and compared with the LPS group

Since monocytes contribute to COVID-CS (Jafarzadeh et al. 2020), the effect of the different Spirulina extracts was also assessed in human monocyte-stimulated cells (Fig. 3a, b). The LED-spirulina extract inhibitory effect was also observed in LPS-stimulated human monocyte, reducing TNF-α levels over 40% at the indicated concentrations.

In addition, the extracts in the absence of LPS did not exhibit off target effect and TNF-α or IL-6 was not detected in non-LPS-stimulated macrophages and monocyte cells.

## Discussion

As reported in previous studies (Feldmann et al. 2020), critically ill COVID-19 patients who were administered a single dose of a TNF-$\alpha$ neutralizing antibody were 45% less likely to die overall, and more likely to be weaning from mechanical ventilation 1 month after treatment, compared with untreated patients. This suggests that control of CS in its early stage through such means as immunomodulators and cytokine antagonists, as well as reduction of lung inflammatory cell infiltration, is key to reducing COVID-19-related mortality rates.

This study showed that LED Spirulina extract in low doses is able to decrease excessive release of TNF-α in LPS-activated macrophages and LPS-activated monocyte cells by over 70% and 40%, respectively. If further clinical trials confirm these efficacy rates among human subjects, then LED Spirulina may act as a novel TNF-α suppressor.

The substantial inhibitory effect of LED Spirulina on TNF-α secretion, in contrast to an unsubstantial effect on IL-6 release, prompts us to assume that the LED Spirulina extract serves as a specific TNF-α suppressor.

It should be emphasized that LED Spirulina’s anti-TNF-α effect exhibited a reversed dose-response activity. This might suggest that very low doses of LED Spirulina extract-related active molecules can affect endpoints (Vandenberg 2014). A similar effect was shown in other plant-based whole extracts, exhibiting the highest activity at the lowest concentration (Otegbade et al. 2017).

A possible explanation for the reaction pattern observed in this study for TNF-α and IL-6 inhibition is that different bioactive molecules of LED Spirulina extract at different absolute quantities affect macrophages and monocyte cells differently, with some bioactive molecules suppressing TNF-α at low dosages, while others inhibit IL-6 release only at higher dosages.

In addition, since both Solar and LED Spirulina extracts include CPC, the cytokine inhibitory bioactivity of the LED Spirulina extract could not be explained by the presence of this molecule alone (Jiang et al. 2017). Furthermore, according to previous studies (Cherng et al. 2007), using pure CPC on LPS-activated macrophages results in 40% TNF-α inhibition at 250 μg/mL. Here, we show inhibitory effect of 70% at 0.1 μg/mL of LED extract, suggesting that CPC is not the only responsible compound for TNF-α inhibition. As there are indications that sorbitol and adenosine derivate have anti-inflammatory properties (Mongkhon et al. 2014), the upregulation of these groups in LED Spirulina compared with Solar Spirulina leads us to suggest that the bioactivity of LED Spirulina extract results from a synergistic effect between several functional groups that might include CPC, sorbitol, and adenosine derivate.

Lastly, in order to preserve a multi-batch consistent bioactivity of the extract, it is essential that the algal biomass is cultivated under consistent and controlled conditions throughout the year (e.g., light composition, irradiation level, temperature, pH levels). Such cultivation conditions may be realized in a fully controlled, indoor system, uninterrupted by diurnal or seasonal variations.

### Implications and Future Research

We suggest at least three advantages of this novel approach of a natural, algae-based CS shield, and note that it requires further research.

First, as TNF-α is a key player in many acute inflammatory reactions, acting as an amplifier of inflammation, algae-based anti-TNF blockade has the potential to assist in treating other virus-induced CS, such as COVID-19, influenza, and autoimmune-related inflammatory diseases.

Second, and on condition that in vivo experiments establish the effectiveness of LED Spirulina in reducing CS, our proposed novel treatment could be dispensed extensively without putting the health of patients at risk, since Spirulina is FDA approved as a dietary supplement, and since its administration is non-intrusive, and can be orally absorbed.

Third, if animal and clinical trials confirm the efficacy of this anti-TNF therapy at the rates reported above, and the substance is available to the general population, a robust therapeutic intervention can be expected that is independent of vaccines, their requisites and potential ramifications, including skilled healthcare workers in clinics and possible side effects, especially in vulnerable populations with chronic diseases, which could only be detected in phase IV clinical trial years after administration.

Concomitantly, an algae-based anti-TNF therapy should be unaffected by variations in SARS-CoV-2 genome across major mutation clusters (Toyoshima et al. 2020), including recent mutations in SARS-CoV-2 that have raised concerns in regard to vaccine efficacy (Korber et al. 2020; Kupferschmidt 2020).

An ancillary, yet substantial benefit, such as therapeutic intervention—should it be proven efficient in human subjects—will free up capacity of ICUs, enable patients to receive over-the-counter affordable treatment option.

Drawing on our in vitro results, we stress the need for prompt in vivo studies to assess the effectiveness of various Spirulina extracts among animal models, as well as in clinical trials.

In addition, future research should note that the effect on TNF-α expression was greater than on IL-6. This finding suggests that the extract targets TNF-α secretion selectively and further validation with a more detailed mechanism of the anti-inflammation cascade within macrophages and monocyte cells should be explored.

It should likewise be noted that the extract from the LED-controlled cultivation process was significantly more effective at suppressing release of TNF-α. To date, no natural compound has been proven to suppress specifically TNF-α. Therefore, LED Spirulina could pave the way for novel algae-based bioactive compounds as anti-TNF treatment.

## Data Availability

Methods, materials, and data used in this study are fully delineated in the text.
